# A novel *BCR-ABL1* mutation in a patient with Philadelphia chromosome-positive B-cell acute lymphoblastic leukemia

**DOI:** 10.2147/OTT.S177019

**Published:** 2018-11-30

**Authors:** Raquel Vinhas, Alexandra Lourenço, Susana Santos, Marcos Lemos, Patrícia Ribeiro, Aida Botelho de Sousa, Pedro Viana Baptista, Alexandra Ramos Fernandes

**Affiliations:** 1 UCIBIO, Life Sciences Department, Faculdade de Ciências e Tecnologia, Universidade Nova de Lisboa, Caparica, Portugal, ma.fernandes@fct.unl.pt; pmvb@fct.unl.pt; 2 Hematology Service, Hospital dos Capuchos (CHLC), Lisbon, Portugal

**Keywords:** ALL, Philadelphia chromosome, e14a2, mutation, p.Y440C

## Abstract

Philadelphia chromosome-positive acute lymphoblastic leukemia (Ph+ ALL) represents the most common genetic subtype of adult ALL (20%–30%) and accounts for approximately 50% of all cases in the elderly. It has been considered the subgroup of ALL with the worst outcome. The introduction of tyrosine kinase inhibitors (TKIs) allows complete hematologic remission virtually in all patients, with improved disease-free survival and overall survival. Nevertheless, the emergence of resistant mutations in BCR-ABL1 may require different TKI strategies to overcome the patient’s resistance and disease relapse. Here, we report a Ph+B-ALL case with persistent minimal residual disease (MRD) after treatment with dasatinib. The patient expressed the P190^BCR-ABL1^ isoform and a novel BCR-ABL1 mutation, p.Y440C. The latter is in the C-terminal lobe of the kinase domain, which likely induces deviations in the protein structure and activity and destabilizes its inactive conformation. The treatment was substituted by bosutinib, which binds to the active conformation of the protein, prior to allogeneic bone marrow transplant to overcome the lack of a complete response to dasatinib. These findings strengthen the importance of BCR-ABL1 mutational screening in Ph+ patients, particularly for those who do not achieve complete molecular remission.

## Introduction

Acute lymphoblastic leukemia (ALL) is a clonal disease in which immature lymphoid cells accumulate mainly in the bone marrow and peripheral blood. The disease is very heterogenous, comprising several hematological, cytogenetic and molecular groups. The Philadelphia chromosome (Ph) is the most frequent genetic abnormality in adult ALL (Ph+ ALL), representing 20%–30% of the B-lineage cases (B-ALL) but only 5% of the pediatric cases. Ph+ ALL frequency increases with age, accounting for approximately 50% of all cases in the elderly.^1^,^2^

The translocation that characterizes the Ph chromosome, t(9;22)(q34;q11), involves the fusion of the following two genes: *BCR* and *ABL1*. In Ph+ ALL, the most frequently expressed isoform of BCR-ABL1, accounting for 70% of the cases, is the P190, in which exon 1 of *BCR* is fused to exon 2 of *ABL1* (e1a2).^3^,^4^ The remaining Ph+ ALL cases are associated with P210^BCR-ABL1^. The transforming potential of the P190 isoform is believed to result from the partial loss of BCR domains, namely, the guanine exchange factor/dbl-like domain that mediates the communication with several Ras proteins, which are crucial for the regulation of signaling pathways and processes, such as cell proliferation, differentiation, adhesion, apoptosis and migration.^4^–^7^ More recently, Reckel et al^8^ demonstrated the existence of strong differences in the interactome and tyrosine phosphoproteome between P190 and P210 signaling pathways. P190 interacts predominantly with the AP2 adaptor complex involved in the regulation of clathrin-mediated endocytosis, whereas P210 interacts mostly with the phosphatase Sts1, showing a stronger activation of the Stat5 transcription factor and the Erk1/2 kinases, while P190 activates the Lyn kinase.^8^ Nonetheless, both transcripts translate for a constitutively active tyrosine kinase that deregulates cell proliferation, differentiation and survival, thus constituting a critical molecular target.

**Figure 1:**
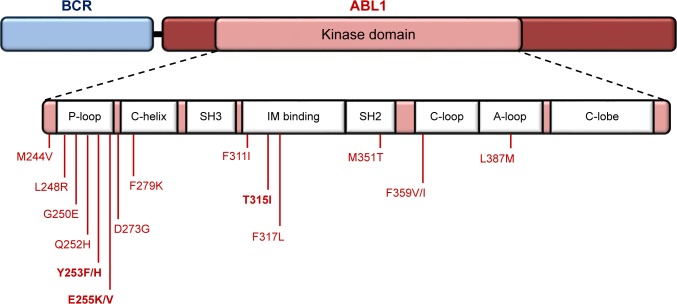
Map of the most recurrent amino acid substitutions in the BCR-ABL1 kinase domain in Ph+ clinical samples. **Notes:** Highlighted mutations are the most frequent, usually associated with IM resistance. Numbering of residues is according to normal ABL1 protein. Key structural motifs within the kinase domain are indicated as follows: P-loop, phosphate binding loop; IM binding, ATP/IM binding region; C-loop, kinase catalytic domain; A-loop, activation loop; C-lobe, C-terminal lobe. **Abbreviations:** IM, imatinib; Ph+, Philadelphia chromosome positive.

In chronic myeloid leukemia (CML), in which all patients test positive for BCR-ABL1, tyrosine kinase inhibitor (TKI) therapy revolutionized prognosis. The same applies for Ph+ALL, in which complete remission can be achieved in 95% of the patients.^4^ Molecular monitoring of Ph+ hematological diseases has become an integral part of the clinical management of these patients, from diagnosis to follow-up surveillance. This is valid not only for transcript identification and quantification (critical for monitoring minimal disease) but also for mutational analysis of the *ABL1* domain of the chimeric transcript. Indeed, BCR-ABL1 variations are one of the mechanisms that lead to TKI resistance, may be due to pre-existent mutations (primary resistance accounts for one-third of the patients) or mutations that arise from TKI treatment pressure (acquired resistance).^9^ For Ph+ ALL patients who fail to respond to imatinib (IM, first-generation TKI), a second- or third-generation TKI (dasatinib, nilotinib, bosutinib and ponatinib) should be administered according to the results of the mutational analysis.^10^ Several studies report the impact of ABL1 mutational screening in Ph+ ALL patients,^11^–^13^ in whom more than 100 mutations have been reported in the ABL1 kinase domain, frequently positioned in the: 1) phosphate-binding loop (P-loop); 2) ATP/IM binding site; 3) catalytic domain (SH2 contact and C-loop); and 4) the activation loop (A-loop) (Figure 1).^11^ IM-resistant Ph+ALL patients frequently harbor the T315I mutation (ATP/IM binding site) and will only respond to ponatinib.^14^,^15^

Herein, we describe a Ph+ B-ALL patient expressing the P190^BCR-ABL1^ isoform and a novel BCR-ABL1 mutation in the region that encodes for the C-terminal lobe domain of the protein.

## Case presentation

**Figure 2:**
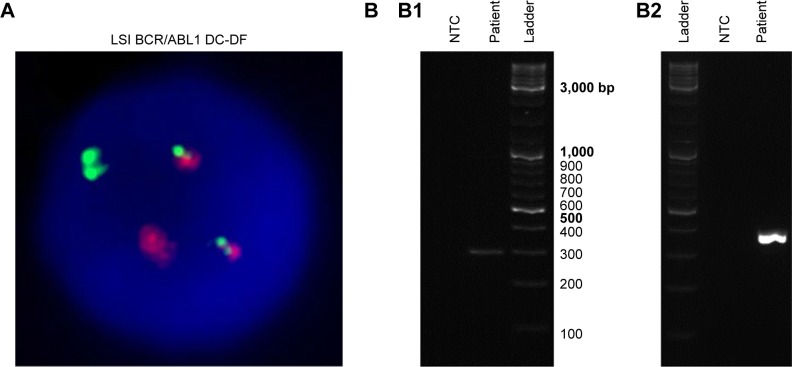
Cytogenetic (**A**) and molecular (**B**) analyses of Ph+ ALL patient’s bone marrow sample. **Notes:** (**A**) Interphase FISH image depicting a Ph+ nucleus, 46,XY,t(9;22)(q34.1;q11.2), using LSI BCR/ABL1 Dual Color, Dual Fusion translocation probe. The image was provided by the Hematology Department of Hospital dos Capuchos. (**B**) *ABL1* and *BCR-ABL1* molecular analyses: (**B1**) control PCR amplification of *ABL1* (expected amplicon size=296 bp) and (**B2**) nested-PCR amplification of e1a2 (P190^BCR-ABL1^) isoform (expected amplicon size=381 bp). PCR products (1 µL) were resolved via 2% agarose gel electrophoresis. Ladder, GeneRuler DNA Ladder Mix SM0331. **Abbreviations:** ALL, acute lymphoblastic leukemia; FISH, fluorescence in situ hybridization; NTC, non-template control; Ph+, Philadelphia chromosome positive.

A 31-year-old male was diagnosed with B-ALL (92% blasts), characterized as Ph+ by cytogenetics (70% fluorescence in situ hybridization [FISH]-positive; Figure 2A; “Methods” section in Supplementary materials). The patient was treated with a pediatric-inspired regimen (Dana Farber Cancer Institute 01-175)^16^ with the addition of dasatinib 140 mg/day. On day 29 of therapy induction, the patient was in complete remission. However, minimal residual disease (MRD) was detected via reverse transcription nested-PCR according to the standardized procedure established by van Dongen et al^17^ (“Methods” section in Supplementary materials and Table S1), which shows a sensitivity level of 10^−4^ for *BCR-ABL1* transcripts (Figure 2B). Molecular analysis of the patient’s white blood cells revealed the expression of P190^BCR-ABL1^ (“Methods” section in Supplementary materials and Figure S1), the most common isoform in Ph+ ALL. MRD was confirmed in a second evaluation 4 weeks later. As per the protocol, this finding triggered a proposal for an allogeneic transplant from a human leukocyte antigen (HLA)-matched sibling.

**Figure 3:**
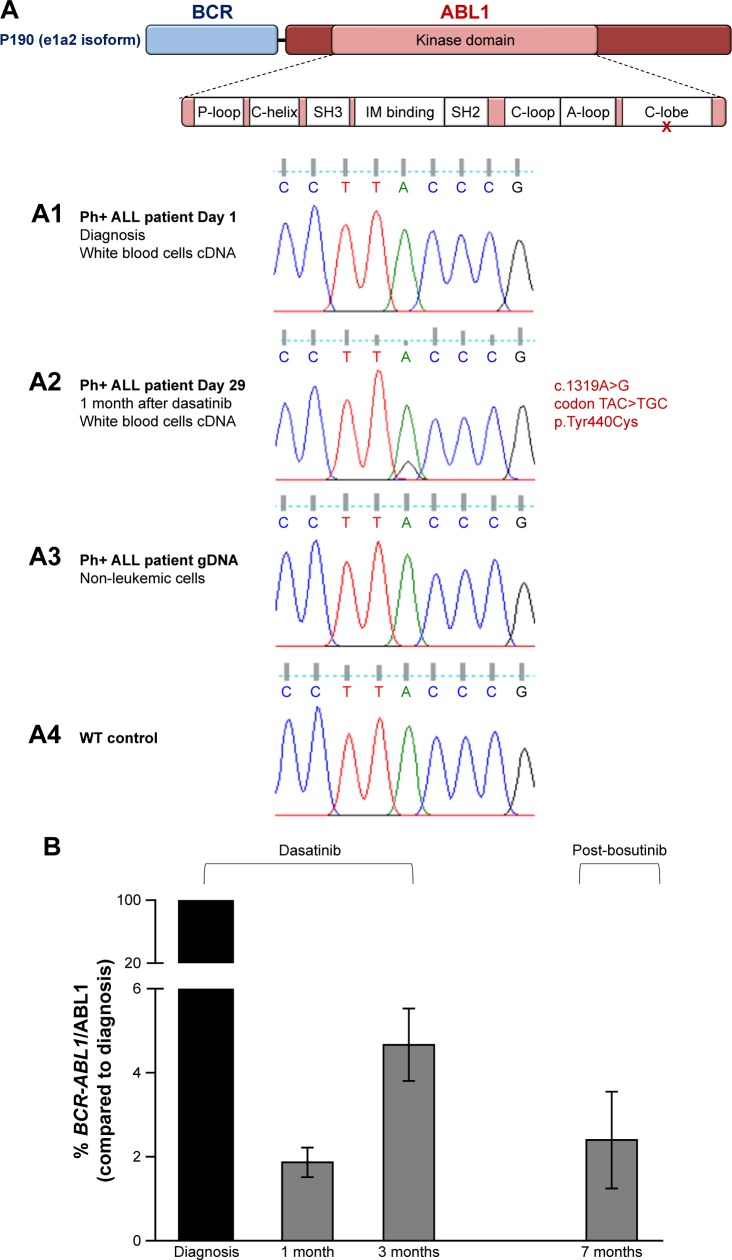
Ph+ ALL patient’s novel BCR-ABL1 point mutation and disease molecular monitoring. **Notes:** (**A**) P190^BCR-ABL1^ protein structure indicating the position of the detected mutation. Sequencing chromatograms displaying: (**A1**) *BCR-ABL1* nucleotide sequence from Ph+ ALL patient’s white blood cells at the time of diagnosis; (**A2**) *BCR-ABL1* nucleotide sequence from Ph+ ALL patient’s white blood cells at day 29 after diagnosis and treatment initiation. Chromatogram peak analysis indicates that the variation is present in 20% of the total Ph+ clones; (**A3**) *ABL1* nucleotide sequence from Ph+ ALL patient’s epithelial cells; and (**A4**) *ABL1* from control individual (WT). P-loop: phosphate binding loop; IM binding: ATP/IM binding region; C-loop: kinase catalytic domain; A-loop: activation loop; and C-lobe: C-terminal lobe. (**B**) *BCR-ABL1* mRNA relative quantification in bone marrow samples at the time of diagnosis and follow-up. qPCR data were normalized to *ABL1* and compared to transcript levels at the time of diagnosis. Patient started induction chemotherapy and dasatinib after diagnosis. Treatment was switched to bosutinib 5 months after diagnosis and maintained for 7 weeks (until the 7-month time point). **Abbreviations:** ALL, acute lymphoblastic leukemia; IM, imatinib; Ph+, Philadelphia chromosome positive; WT, wild-type.

Bone marrow samples were further analyzed for mutations associated with TKI resistance so as to reduce *BCR-ABL1* expression before the allogeneic bone marrow transplant. Mutational analysis via Sanger sequencing (“Methods” section in Supplementary materials) showed a point mutation in the *ABL1* domain of the fusion transcript, c.1319A.G, which lies in the region that translates for the C-terminal lobe of the kinase domain, p.Tyr440Cys (Figure 3). Despite this variation being present in 20% of the *BCR-ABL1*-expressing cells at follow-up (day 29; Figure 3A2), it had not been identified at diagnosis (day 1; Figure 3A1). These results indicate that this might be acquired resistance probably due to selective pressure on mutant clones upon dasatinib treatment. In fact, the in-frame mutation in leukemic cells is not present in *ABL1* from non-leukemic cells, indicating a somatically acquired rather than a germline mutation. This was confirmed by the analysis of the genomic DNA of the patient’s epithelial cells (Figure 3A3).

Following extensive bioinformatics and literature analyses (discussed in the “Discussion” section), and as an effort to achieve molecular remission, the TKI regimen was changed to bosutinib in post-induction therapy. After 7 weeks on bosutinib, *BCR-ABL1* levels did decrease when compared to those on dasatinib (Figure 3B). Nevertheless, MRD was still present, and thus the patient proceeded to transplant. He died 7 months posttransplant from complications of acute graft vs host disease.

## Discussion

TKI regimens have greatly improved the therapeutic outcome of patients with Ph+ ALL. However, each individual’s sensitivity to each TKI must be considered for a successful therapeutic outcome. Similar to what is recommended for CML, Ph+ ALL patients who do not respond to one of the available TKIs should undergo mutational screening for an assessment of markers of drug resistance mechanisms.^4^,^12^

The herein newly described *BCR-ABL1* nucleotide substitution, c.1319A.G (codon TAC.TGC), results in the amino acid alteration, p.Tyr440Cys (Figure 3). The substitution of a highly conserved amino acid across different species (Table S2), coupled to a shift between a large amino acid (tyrosine) and a small amino acid with a thiol side chain (cysteine), is likely to induce deviations in correct folding of the protein. To understand the possible effect of this mutation in BCR-ABL1 structure, we probed the sequence through MutationTaster, which predicts changes in the transcript splice sites, as well as alterations to protein features.^18^ Using Support Vector Machine and sequence information, a decrease in the stability of protein structure due to the p.Tyr440Cys mutation is predicted with a confidence score of −0.898.^19^ DISOclust software analysis also shows an increase in the disorder probability score in the mutated ABL1 domain, particularly in residues located after the Y440C mutation (Figure S2).^20^

Point mutations within the C-terminal lobe region of BCR-ABL1 have been described to impair protein structure. For example, the well-studied E459K substitution makes the protein inactive conformation unstable even though the amino acid position is not near the ATP/IM binding site. Since IM only binds to the inactive conformation of BCR-ABL1, patients harboring this mutation typically do not respond to this TKI or other first-line TKIs, but are more sensitive to bosutinib that can bind to the active conformation of the protein.^21^ On this basis, the treatment was altered to bosutinib, which induced a reduction in MRD after 7 weeks (Figure 3B), to set up for allogeneic transplant.

To the best of our knowledge, this is the first report regarding the c.1319A.G variation in the *ABL1* domain of *BCR-ABL1* of a refractory Ph+ patient to dasatinib treatment. The predicted change in protein features is likely to induce resistance to TKIs and should be carefully addressed upon during treatment choice.

## Ethical approval and consent

Written informed consent to have the case details published was obtained, and the study was approved by Hospital dos Capuchos Ethics Committee. All approved ethical requirements for sample collection and assortment, processing and analysis required by the ethics committee have been strictly followed.

## Supplementary materials

### Methods

#### FISH analysis

FISH analysis was performed in interphase cells using the LSI BCR/ABL1 Dual Color, Dual Fusion translocation probe (Abbott Molecular, Des Plaines, IL, USA), according to the manufacturer’s instructions. A total of 100 nuclei were scored at diagnosis. This analysis was carried out using a fluorescence microscope (Eclipse Ci; Nikon, Tokyo, Japan), and image capture was performed using Isis FISH Imaging System V 5.7.1 (MetaSystems, Altlussheim, Germany).

#### RNA extraction and cDNA synthesis

RNA was extracted from bone marrow white blood cells. Briefly, 5 mL of sample was mixed with 10 mL of red blood cell lysis solution (5 mM MgCL_2_, 10 mM NaCl and 10 mM Tris-HCl; pH 7) and centrifuged at 400× *g* for 5 minutes. The procedure was repeated to lyse residual red blood cells. White blood cell pellet was subjected to total RNA extraction using SV Total RNA Isolation System (Promega Corporation, Fitchburg, WI, USA), according to the manufacturer’s instructions. RNA was resuspended in nuclease-free water and stored at −80°C until use. RNA concentration and purity were determined by UV spectrophotometry. Total RNA extracted (100 ng) was reverse transcribed into cDNA, using the NZY M-MuLV First-Strand cDNA Synthesis kit (Nzytech, Lisbon, Portugal).

#### BCR-ABL1 molecular analysis

Nested-PCR amplification of *BCR-ABL1* was performed using primers specified in Table S1. The PCR mixture components included 1× NH_4_ reaction buffer (Bioline, Taunton, MA, USA), 2.5 mM MgCl_2_, 0.15× Hi-Specific additive (Bioline), 200 µM dNTPs, 400 nM primers and 0.02 U/µL BIOTAQ DNA polymerase (Bioline). Outer PCR was performed using 3 µL of cDNA in a 50 µL reaction, under the following conditions: initial denaturation at 95°C for 5 minutes; 30 cycles of 94°C for 30 seconds, 55°C for 30 seconds, 72°C for 1.5 minutes; and a final extension step at 72°C for 10 minutes. Inner PCR was performed using 1 µL of outer PCR product in a 50 µL reaction, under the following conditions: initial denaturation at 95°C for 5 minutes; 30 cycles of 94°C for 15 seconds, 55°C for 30 seconds, 72°C for 1 minute; and a final extension step at 72°C for 10 minutes. A control PCR, for the amplification of *ABL1*, was also performed using primers depicted in Table S1. All PCR products were visualized via 2% agarose gel electrophoresis and subsequently analyzed by Sanger sequencing at STABVIDA (Setúbal, Portugal).

#### Relative quantification of BCR-ABL1 transcripts via qPCR

Quantitative monitoring of *BCR-ABL1* e1a2 transcript was performed in bone marrow samples using a Corbett Rotor-Gene 6000 thermal cycler (Qiagen NV, Venlo, the Netherlands) and NZY qPCR Green Master Mix (Nzytech). The reaction mixture was prepared in a final volume of 10 µL with 1 µL of cDNA and 100 nM of each primer: outer PCR primers for *BCR-ABL1* transcript identification or *ABL1* control primers, specified in Table S1. qPCR conditions included an initial denaturation at 95°C for 15 minutes and 40 cycles of 95°C for 20 seconds and 60°C for 50 seconds. qPCR data were analyzed by the Ct method (2^−ΔΔCt^), in which relative gene expression is given by quantification of the gene of interest (*BCR-ABL1*) normalized to internal control gene (*ABL1*), relative to control condition (diagnostics sample).

#### Genomic DNA extraction from patient’s epithelial cells

Buccal epithelial cells were collected from the subject of this study by twirling a swab on the inner cheeks for 1 minute. The swab was separated from the stick and inserted into a microtube containing 440 µL of lysis buffer (50 mM Tris; 1% SDS). The tube was vortexed for several minutes to promote the release of most epithelial cells. The swab was removed, and 20 µL of proteinase K (100 mg/mL dissolved in TE buffer: 10 mM Tris-HCl; 0.1 mM EDTA pH 8) was mixed with the cell suspension and incubated for 10 minutes at 50°C. After adding 40 µL of 5 M NaCl, 500 µL of isopropanol was gently mixed with the DNA-containing solution and allowed to stand for 5 minutes at room temperature. The sample was centrifuged for 5 minutes at 12,000× *g*; the supernatant was removed; and the dry pellet was resuspended in 100 µL of TE buffer. DNA concentration and purity were determined by UV spectrophotometry. DNA was stored at 4°C until use.

#### Mutational analysis of ABL1 exon 8 on genomic DNA

PCR amplification of *ABL1* exon 8 of patient’s epithelial cells was performed using the following primers: forward 5′-CTCAAATAATCCTCCCACTTCA and reverse 5′-CCTGGAATGCCCACATATAC. PCR mixture components included 1× NH_4_ reaction buffer (Bioline), 2.5 mM MgCl_2_, 0.15× Hi-Spec additive (Bioline), 200 µM dNTPs, 400 nM primers, 0.02 U/µL BIOTAQ DNA polymerase (Bioline) and 20 ng of gDNA. PCR conditions included an initial denaturation at 95°C for 5 minutes; 30 cycles of 94°C for 30 seconds, 55°C for 30 seconds, 72°C for 1 minute; and a final extension step at 72°C for 10 minutes.

#### Sequence alignments

Basic Local Alignment Search Tool (BLAST), from the National Center for Biotechnology Information (NCBI), was used to align all obtained sequences, via Sanger sequencing, with the following reference sequences: *BCR-ABL1* e1a2 (GenBank AF113911.1) and *ABL1* (GenBank NM005157.5).

Figure S1 Alignment of Ph+ ALL patient e1a2 nucleotide sequence (Seq 1) with *BCR-ABL1* e1a2 reference sequence from GenBank AF113911.1 (Seq 2).
**Note:** Arrow indicates the breakpoint in this *BCR-ABL1* isoform.
**Abbreviations:** ALL, acute lymphoblastic leukemia; Ph+, Philadelphia chromosome positive.

Figure S2 DISOclust disorder prediction results.
**Note:** Disorder probability score by residue number in BCR-ABL1 harboring the Y440C on the ABL1 domain.

**Table S1:** Nested-PCR primers for the amplification of *ABL1*, *BCR-ABL1* transcript identification and analysis of *BCR-ABL1* mutational status

	ABL1 – control	Transcript identification	Mutational status	
Outer PCR	For 5′-GGCCAGTAGCATCTGACTTTG (ABL1 exon 2) Rev 5′-ATGGTACCAGGAGTGTTTCTCC (ABL1 exon 3)	For 5′-GACTGCAGCTCCAATGAGAAC (BCR exon 1) Rev 5′-GTTTGGGCTTCACACCATTCC (ABL1 exon 3)	For 5′-CAGAACTCGCAACAGTCCTTC (BCR exon 1) Rev 5′-CTTCGTCTGAGATACTGGATTCCT (ABL1 exon 9)	
Inner PCR	N/A	For 5′-CAGAACTCGCAACAGTCCTTC (BCR exon 1) Rev 5′-TTCCCCATTGTGATTATAGCCTA (ABL1 exon 3)	For 5′-GCAACAAGCCCACTGTCTAT (ABL1 exon 4) Rev 5′-TGTTGTAGGCCAGGCTCTC (ABL1 exon 7)	For 5′-TGAGCAGGTTGATGACAGG (ABL1 exon 7) Rev 5′-TGAGATACTGGATTCCTGGAAC (ABL1 exon 9)

**Abbreviations:** For, forward; N/A, not applicable; Rev, reverse.

**Table S2:** ABL1 protein alignment between different species demonstrates the conservation of the affected amino acid

Species	AA position	Alignment
*Homo sapiens* mutated (Ph+ ALL patient)	440	LWEIATYGMSPCPGIDLSQVYEL
*H. sapiens* wild-type	440	LWEIATYGMSPYPGIDLSQVYEL
*Pan troglodytes*	459	LWEIATYGMSPYPGIDLS
*Macaca mulatta*	168	LWEIATYGMSPYPGIDLSQVYEL
*Felis catus*	459	LWEIATYGMSPYPGIDLS
*Mus musculus*	459	LWEIATYGMSPYPGIDLS
*Gallus gallus*	440	LWEIATYGMSPYPGIDLSQVYEL
*Takifugu rubripes*	801	LWEIATYGMSPYPGIDL
*Danio rerio*	459	LWEIATYGMSPYPGIDLSQ
*Drosophila melanogaster*	587	LWEIATYGMSPYPAIDLTDVYHK
*Caenorhabditis elegans*	509	LWEIATYGMAPYPGVELSNVYGL

**Abbreviations:** ALL, acute lymphoblastic leukemia; Ph+, Philadelphia chromosome positive.
