# Supplementary figures and images for: A novel BCR-ABL1 mutation in a patient with Philadelphia chromosome-positive B-cell acute lymphoblastic leukemia
Source: Onco Targets Ther. 2018 Nov 30;11:8589–98. doi: 10.2147/OTT.S177019 (PMC6280987; doi:10.2147/OTT.S177019)

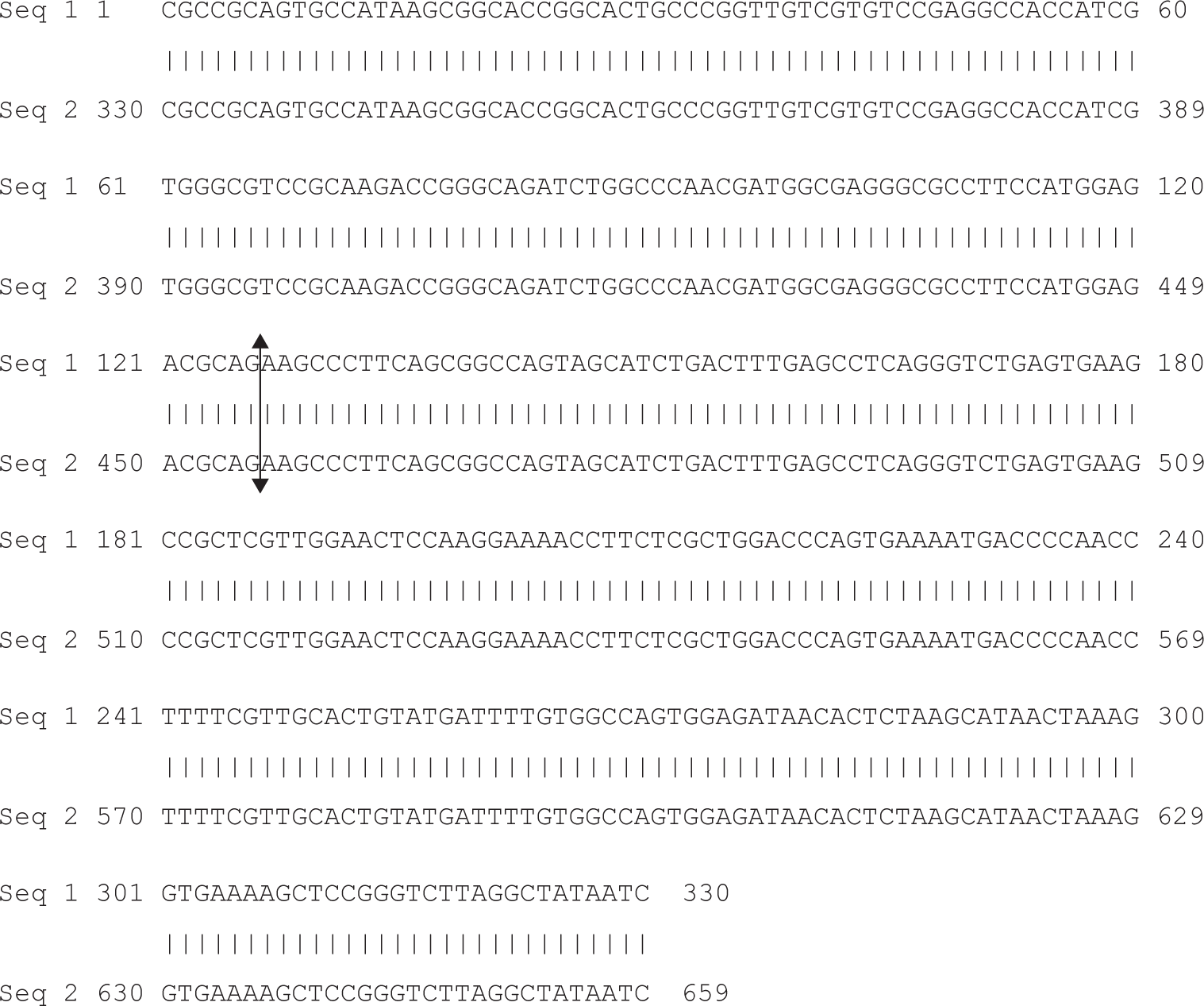

Supplement: Figure S1 — Alignment of Ph+ ALL patient e1a2 nucleotide sequence (Seq 1) with BCR-ABL1 e1a2 reference sequence from GenBank AF113911.1 (Seq 2). Note: Arrow indicates the breakpoint in this BCR-ABL1 isoform. Abbreviations: ALL, acute lymphoblastic leukemia; Ph+, Philadelphia chromosome positive. [file ott-11-8589s1.tif]

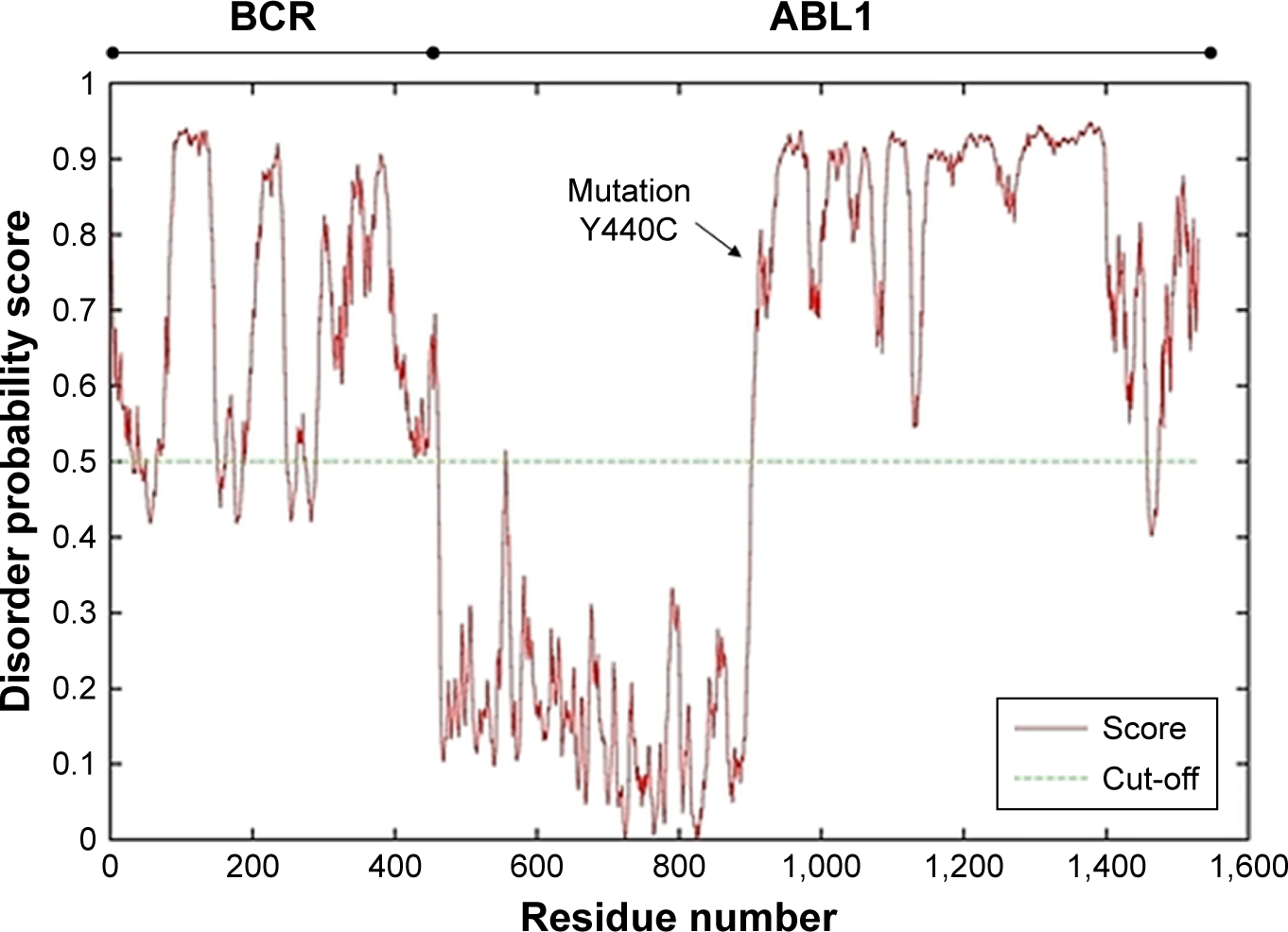

Supplement: Figure S2 — DISOclust disorder prediction results. Note: Disorder probability score by residue number in BCR-ABL1 harboring the Y440C on the ABL1 domain. [file ott-11-8589s2.tif]
